# P-2256. Clinical characteristics and outcomes of non-typhoidal Salmonellosis in patients with hematologic malignancy

**DOI:** 10.1093/ofid/ofae631.2409

**Published:** 2025-01-29

**Authors:** Cesar G Berto, Jessica S Little, Sophia Koo, Elizabeth Hohmann, Sarah P Hammond

**Affiliations:** The University of Alabama at Birmingham, Birmingham, AL; Brigham and Women's Hospital, Boston, Massachusetts; Brigham and Women's Hospital, Dana-Farber Cancer Institute, Boston, MA; Massachusetts General Hospital, Boston, Massachusetts; Massachusetts General Hospital, Boston, Massachusetts

## Abstract

**Background:**

Non-typhoidal salmonellosis is a significant cause of foodborne illness in the US, especially in immunocompromised host. Previous studies report a higher incidence in cancer patients; however, advancements in cancer therapy and rising antibiotic resistance have reshaped this association. Salmonellosis in patients with hematologic malignancy (HM) remains poorly characterized.

Characteristics of HM patients with non-typhoidal Salmonella infection

**Figure:**
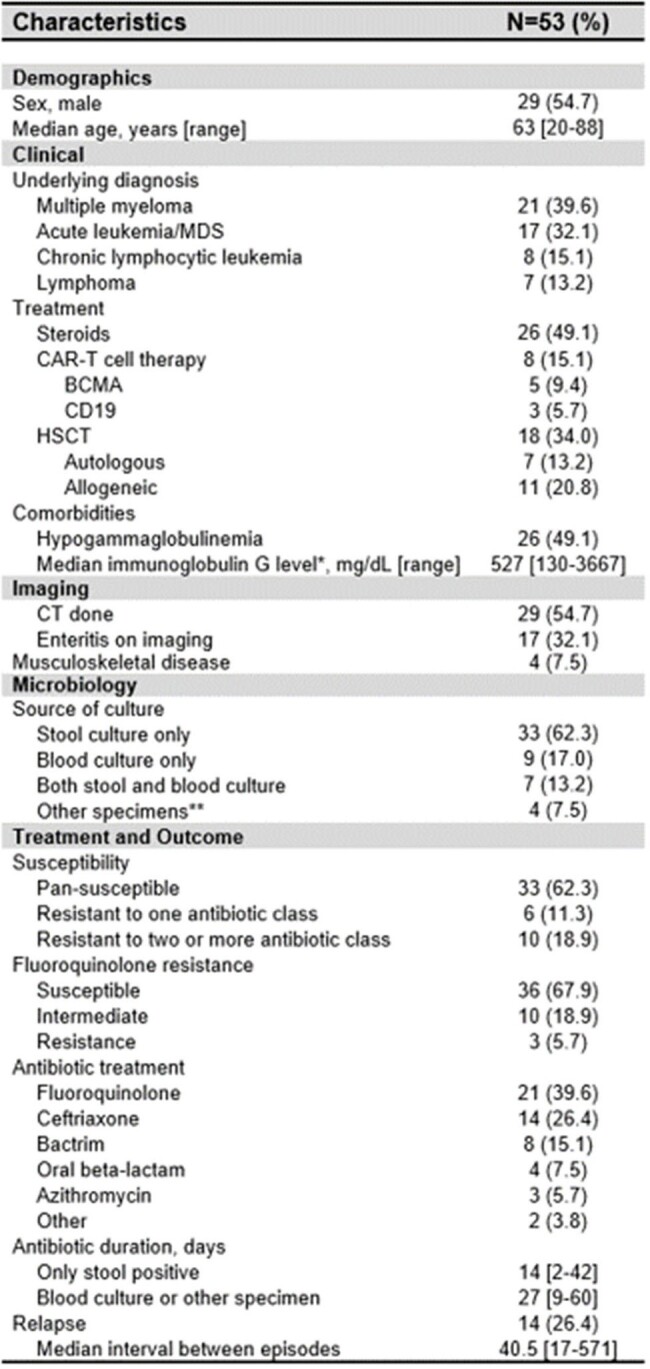

*Only for those who were not receiving intravenous immunoglobulin therapy

**Included culture from splenic abscess, lymph node, soft tissue, and bone

**Methods:**

We identified non-typhoidal salmonella infections in HM patients from 1/1/2016 to 12/31/2023 at 2 institutions. Clinical, microbiologic and treatment outcomes were collected. Hypogammaglobulinemia (HGG) was defined as having immunoglobulin G (IgG) level < 500 IU/dL or receiving intravenous immunoglobulin for past diagnosis of low IgG. Relapse was defined as confirmed *Salmonella* infection with symptoms recurring after clinical resolution with an initial antibiotic course. Chi square and Mann-Whitney U tests were used to examine the relationship between clinical parameters and relapse.

**Table 2.**
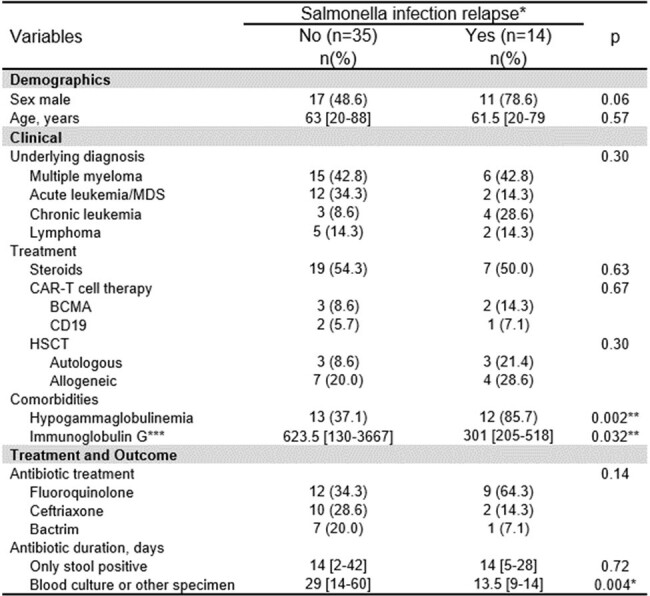
Demographic and clinical characteristics of HM patients by relapse of Salmonella infection

* Four patients did not have enough follow-up to determine relapse

** p<0.05

*** Only for those who were not receiving intravenous immunoglobulin therapy

**Results:**

We identified 53 HM patients with non-typhoidal salmonellosis. Clinical and microbiological variables are shown in Table1. Most individuals were male (54.7%) with median age of 63 years [20-88]. Multiple myeloma was the most common diagnosis (39.6%), followed by acute leukemia/myelodysplastic syndrome (32.1%); 18 patients underwent hematopoietic cell transplantation and 8 patients received chimeric antigen T-cell therapy. Twenty-six patients (45.3%) had HGG. Salmonella was isolated from stool cultures (62.3%), blood cultures (17%), both (13.2%) or another specimen (7.5%). Four patients (7.5%) had musculoskeletal involvement. Relapse occurred in 14 cases (26.4%). In a bivariate analysis (Table 2), HGG was associated with relapse (p=0.002) and those with relapse had a lower median IgG level (p=0.032). Among patients with extraintestinal infection, those who experienced relapse received a shorter initial duration of antimicrobials to those without relapse (p=0.004; figure 1).

Duration of antibiotic therapy in days for patients with extra-intestinal salmonella infection

**Figure:**
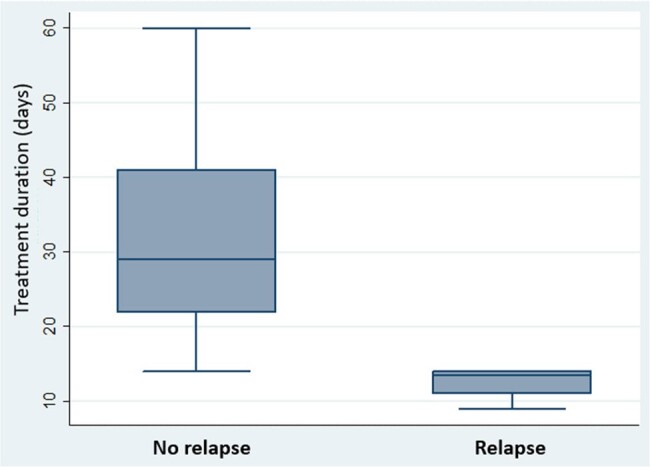

**Conclusion:**

HM patients are at high risk for non-typhoidal salmonellosis. HGG and shorter course of antibiotics are associated with an increased relapse risk, particularly in those with extraintestinal involvement. Extended duration of antibiotic therapy could be considered for these high-risk patients.

**Disclosures:**

Elizabeth Hohmann, MD, Astra Zeneca: Grant/Research Support|Laguna Biotherapeutics: Grant/Research Support|MicrobiomeX/Tend: Grant/Research Support Sarah P. Hammond, MD, Cidara: Grant/Research Support|F2G: Grant/Research Support|GSK: Grant/Research Support|Melinta: Advisor/Consultant|Pfizer: Advisor/Consultant|Roche: Advisor/Consultant|Scynexis: Grant/Research Support|Seres Therapeutics: Advisor/Consultant

